# Structure of Polysaccharide from *Dendrobium nobile* Lindl. and Its Mode of Action on TLR4 to Exert Immunomodulatory Effects

**DOI:** 10.3390/foods13091356

**Published:** 2024-04-28

**Authors:** Lian Li, Hang Chen, Guichun Huang, Yiyi Lv, Li Yao, Zhongxia Guo, Shuyi Qiu, Xiaodan Wang, Chaoyang Wei

**Affiliations:** 1 Key Laboratory of Fermentation Engineering and Biological Pharmacy of Guizhou Province, School of Liquor and Food Engineering, Guizhou University, Guiyang 550025, China; lilian552022@163.com (L.L.); chenhang46@outlook.com (H.C.); 18386244213@163.com (G.H.); lvyi1891@163.com (Y.L.); liyao565@163.com (L.Y.); 18702424389@163.com (Z.G.); syqiu@gzu.edu.cn (S.Q.); wangxiaodan0516@126.com (X.W.); 2 Key Laboratory of Plant Resource Conservation and Germplasm Innovation in Mountainous Region (Ministry of Education), College of Life Sciences/Institute of Agro-Bioengineering, Guizhou University, Guiyang 550025, China

**Keywords:** *Dendrobium nobile* Lindl. polysaccharide, structural characterization, TLR4-MD2 complex, anti-inflammatory

## Abstract

*Dendrobium nobile* Lindl. polysaccharide (DNP1) showed good anti-inflammatory activity in our previous study. In this study, the structural characterization of DNP1 and its mode of action on TLR4 were investigated. Structural characterization suggested that DNP1 was a linear glucomannan composed of (1 → 4)-β-Man*p* and (1 → 4)-β-Glc*p* residues, and the acetyl group was linked to the C-2 of Man*p*. The possible repeating structural units of DNP1 were [→4)-2-OAc-β-Man*p*-(1→]_3_ →4)-β-Glc*p*-(1→. Surface plasmon resonance (SPR) binding test results showed that DNP1 did not bind directly to TLR4. The TLR4 and MD2 receptor blocking tests confirmed that DNP1 needs MD2 and TLR4 to participate in its anti-inflammatory effect. The binding energy of DNP1 to TLR4-MD2 was −7.9 kcal/mol, indicating that DNP1 could bind to the TLR4-MD2 complex stably. Therefore, it is concluded that DNP1 may play an immunomodulatory role by binding to the TLR4-MD2 complex and inhibiting the TLR4-MD2-mediated signaling pathway.

## 1. Introduction

*Dendrobium nobile* Lindl. is a traditional and precious Chinese herbal medicine with pharmacological activities, such as anti-tumor, hypoglycemic, and anti-aging [1,2]. Its chemical constituents mainly include alkaloids, flavonoids, coumarins, phenols, polysaccharides, etc. [3]. *Dendrobium nobile* Lindl. polysaccharides (DNPs) are rich in content and have many biological activities, such as anti-tumor [2,4], antiviral [5], antioxidant [6], and immune activity [7]. The molecular weight of DNPs is 2.55 KDa-770 KDa [8,9]. The monosaccharide composition includes glucose, mannose, galactose, arabinose, xylose, rhamnose, galacturonic acid, etc. The types of glycosidic bonds are α-(1 → 4), α-(1 → 6), β-(1 → 4), β-(1 → 6), etc. [10,11].

Our previous study found that ultrasonically extracted DNP1 has good anti-inflammatory activity, significantly reducing NO and pro-inflammatory cytokine levels in lipopolysaccharide-stimulated macrophages [12]. Inflammation is an innate immune response induced by infection or injury and is an adaptive response of the body to harmful diseases. However, in the absence of infection or obvious tissue damage, tissue stress and dysfunction can also induce inflammation, and excessive inflammatory reactions can cause damage to the body [13,14]. Pro-inflammatory cytokines, such as TNF-α, IL-1β, and IL-6, play an irreplaceable and important role in the regulation of inflammation as a dynamic process [15]. TNF-α elicits and transduces intracellular inflammatory signals that can induce cell necrosis and apoptosis and stimulate macrophages to release pro-inflammatory cytokines, such as IL-6 and IL-1β [16,17]. IL-6 and IL-1β are also two very important inflammatory cytokines, similar in nature and function to TNF-α, which are responsible for acting on monocytes and macrophages, as well as being responsible for a series of intracellular signaling events that can enhance immune function [18]. However, overexpression of TNF-α, IL-1β, and IL-6 is harmful to the human body [19].

Lipopolysaccharide (LPS) constitutes a significant component of the outer membrane of Gram-negative bacteria and stimulates macrophages to generate inflammatory factors and NO, hence initiating an inflammatory response [20]. TLR4 serves as the primary LPS receptor on the surface of monocytes, macrophages, and dendritic cells, facilitating innate immunity [21]. TLR4 mediates the phagocytic inflammatory response to various microorganisms by activating the NF-κB signaling pathway to induce the inflammatory response [22,23]. Recognition of LPS requires TLR4 and MD2 to form the TLR4-MD2 complex, and then the TLR4-MD2 complex combines with LPS to mediate signal transduction and stimulate macrophages to produce an inflammatory response [17]. Many natural compounds have been reported to combine with TLR4-MD2 to exert pro-inflammatory or anti-inflammatory activities [24,25]. Therefore, we speculate that DNP1 could bind to TLR4-MD2 instead of LPS and block the TLR4-MD2-mediated NF-κB/MAPK signaling pathway, thus exerting its immunomodulatory effect.

In our previous work, the ultrasonic extraction process of DNP1 was optimized and found to have good anti-inflammatory activity [12], but the chemical structure of DNP1 and the mechanism of its anti-inflammatory activity are still unknown. In this study, methylation analysis and 1D/2D NMR were used to characterize the structure of DNP1 and to characterize the structural units and the chain conformation of DNP1. The surface plasmon resonance (SPR) binding assay, simulated molecular docking, and macrophage receptor blocking assay were used to explore the binding mode of DNP1 and TLR4.

## 2. Materials and Methods

### 2.1. Materials and Reagents

*Dendrobium nobile* Lindl. was obtained from Guizhou Chishui Guoli *Dendrobium nobile* Lindl. Development Co., Ltd. (Guizhou, China), dried and crushed, and then screened with 80 meshes.

MD2-IN-1, TLR4-IN-C34, IL-10, IL-6, IL-1 β, TNF-α ELISA kit were purchased from Shanghai Jianglai Biotechnology Co., Ltd. (Shanghai, China); mouse macrophage RAW264.7 was provided by Zhejiang Meisen Cell Technology Co., Ltd. (Wenzhou, China); DMEM High-Glucose Medium and FBS were acquired from Gibco (New York, NY, USA); CCK-8 Kit was purchased from Wuhan Doctor De Bioengineering Co., Ltd. (Wuhan, China); NO detection kit was obtained from Shanghai Biyuntian Biotechnology Co., Ltd. (Shanghai, China); trifluoroacetic acid, acetic acid, LPS, dimethyl sulfoxide, sodium borodeuteride, and acetic anhydride were purchased from Sigma (Kalamazoo, MI, USA).

### 2.2. Molecular Weight and Conformational Determination of DNP1

Next, 1 mg of DNP1 was accurately weighed and dissolved in the 1 mL mobile phase, and the solution was filtered through a filter membrane with a pore size of 0.45 μm before being tested. The instrument model and parameters were obtained from our previous reports [12]. The chromatographic system had the following elution conditions: column temperature 45 °C, injection volume 100 μL, mobile phase of 0.1 mol/L NaNO_3_ solution containing 0.02% NaN_3_, flow rate 0.4 mL/min, and isocratic elution for 100 min.

### 2.3. Methylation Determination

The DNP1 linkage patterns were studied by methylation and GC–MS analysis according to Ciucanu and Kerek [26] and the previous method [27]. Briefly, 3 mg of DNP1 was added to 500 μL well-prepared anhydrous NaOH-DMSO solution. Then, 50 μL of CH_3_I was added to the above solution, mixed well, and allowed to react for 1 h. The reaction was terminated by adding 1 mL of distilled water. The resulting solution was then mixed with 2 mL of dichloromethane, centrifuged, and the aqueous phase was discarded. The organic solution was washed three times with distilled water and dried. The methylated products were then depolymerized, hydrolyzed, reduced, acetylated, and analyzed with a gas chromatography mass spectrometer (GC–MS, Agilent 7890 A/5977C, Santa Clara, CA, USA).

### 2.4. NMR Analysis

Next, 30 mg of DNP1 was dried with P_2_O_5_ in a desiccator for two days and then dissolved in 1 mL of D_2_O. After lyophilization, it was re-dissolved in 1 mL of D_2_O, and this process was repeated twice. Hydrogen protons were exchanged in the process. Finally, it was dissolved in 1 mL of D_2_O and transferred to an NMR tube; tetramethylsilane (TMS) was used as an internal standard. NMR spectra (^1^H NMR, ^13^C NMR, NOESY, COSY, HSQC and HMBC) were recorded at 25 °C using a Bruker Advance 600M spectrometer (Bruker, Rheinstetten, Germany). All chemical shifts are expressed in ppm, and the chemical shift of TMS in D_2_O is 0.00/0.00 ppm (^13^C/^1^H).

### 2.5. Surface Plasmon Resonance (SPR) Binding Test

The binding affinity of DNP1 to the target TLR4 protein was determined in the Biacore T200 system (GE Healthcare, Pittsburgh, PA, USA) according to the following procedure: (1) Immobilization of anti-histidine antibody (Anti-His, LOT# 10313498, provided by Sanyou Biopharmaceuticals Co., Ltd., Shanghai, China) to the chip: anti-histidine antibody was diluted to 50 μg/mL with acetate (pH 4.5), and 1–4 channels of the CM5 chip (GE Healthcare, Pittsburgh, PA, USA) were selected for the capture experiment. (2) Capture the ligand (His tag) on the chip: the antigen (His tag) was diluted to 1000 nM with 1 × HBS-EP (pH 7.4) buffer. After setting the flow rate at 10 μL/min, we selected 3 channels of the His chip to capture the receptor experiment. (3) Dynamic parameter setting 1 × HBS-EP buffer (pH 7.4) was used to adjust DNP1 to 10,000 nM, and then diluted to 5000, 2500, 1250, 625, 312.5, 156.25, 78.125 nM; when the flow rate was 30 μL/min, the antigen (hu-TLR4-his, 70.68 kDa, provided by Sanyou Biopharmaceuticals Co., Ltd., Shanghai, China) was captured on the chip for 30 s, combined with DNP1 (various concentration) for 180 s, dissociated in buffer for 300 s, and the reaction temperature was 25 °C.

### 2.6. Molecular Docking

The three-dimensional structure of DNP1 was drawn by Discovery Studio 2019 with total atomic energy minimization. AutoDockTools was used to set up a series of settings, such as hydrogenation, charge calculation, charge distribution, etc., to obtain a file in the “pdbqt” format of DNP1. The crystal structure of TLR4-MD2 (PDB ID: 2Z64) was obtained from the protein database at http://www.rcsb.org/ (accessed on 9 July 2023). The protein was then imported into the PyMOL software to remove the initial ligand and water molecules. Hydrogenation, charge calculation, and charge distribution of TLR4-MD2 were obtained as “pdbqt” format files using AutoDockTools. The center coordinates were set to x = −30.37, y = −5.82, and z = 9.4, and the dimensions of the grid dot box were set to 40 × 40 × 40. All other parameters were left in their default settings. AutoDock Vina was adopted to investigate the interactions between DNP1 and TLR4-MD2 and their binding affinity.

### 2.7. TLR4 and MD2 Receptor Blocking Test

In this experiment, TLR4-IN-C34 and MD2-IN-1 were used to block the TLR4 and MD2 proteins, LPS was used to induce an inflammatory reaction, and then DNP1 was added to study the relationship between the anti-inflammatory activity of DNP1, TLR4, and MD2.

#### 2.7.1. Cell Culture

RAW 264.7 mouse macrophages were taken in a logarithmic growth phase, and the concentration of cell suspension was adjusted to 1 × 10^5^ cells/mL and inoculated in 96-well plates. TLR4-IN-C34 (100 μM) or MD2-IN-1 (100 μM) was added to the plates and then incubated at 37 °C for 24 h in a cell culture incubator containing 5% CO_2_. After cell attachment, culture medium was replaced with serum-free DMEM medium and 1 μg/mL LPS. DNP1 was dissolved in DMEM solution with a concentration gradient of 12.5 μg/mL, 25 μg/mL, 50 μg/mL, 100 μg/mL, and 200 μg/mL. The cells were then added to 50 μL of DNP1 and incubated overnight in a cell culture incubator at 37 °C. Under identical conditions, the DNP1 solution was replaced with DMEM in the control group; the cell suspension and DNP1 solution were replaced with DMEM in the blank group.

#### 2.7.2. Detection of Cell Proliferation and Cytotoxicity

The CCK-8 assay was used to detect the proliferation and cytotoxic activity of RAW 264.7 mouse macrophages after the addition of different concentrations of DNP1 solution. The CCK-8 solution was thoroughly blended with serum-free DMEM medium in a volume ratio of 1:10 prior to beginning the assay. The cell culture operation was conducted as mentioned above. The RAW 264.7 mouse macrophages were removed after culturing in the specific medium, and the cells were washed with PBS solution. The operation was repeated twice, and the prepared CCK-8 mixture was added to ensure coverage of each sample. After incubation at 37 °C for three hours, 100 µL of the solution was extracted from each well. Absorbance values were measured at 450 nm using an enzyme labeling device (Labsystems Multiskan MS 352, Vantaa, Finland). Cell viability was calculated according to the following formula:

Cell survival rate (%) = absorbance value of the treatment group/absorbance value of the control group × 100%

#### 2.7.3. Detection of Inflammatory Cytokines

The supernatant culture solution after incubation was taken and centrifuged at 4 °C for 10 min at 1000 r/min. And the supernatants were added to the commercial kit to determine the NO content. The cytokines content (including TNF-a, IL-1β, and IL-6) was measured using Elisa kits. For specific operations, please refer to the instruction manual.

### 2.8. Statistical Analysis

Three replicates were set for each experiment, and the data were expressed as average ± standard deviation. Significant differences (*p* < 0.05) were evaluated by one-way analysis of variance (ANOVA) in a completely randomized design and analyzed using SPSS 21.0 software.

## 3. Results

### 3.1. Molecular Weight and Conformation of DNP1

**Figure 1:**
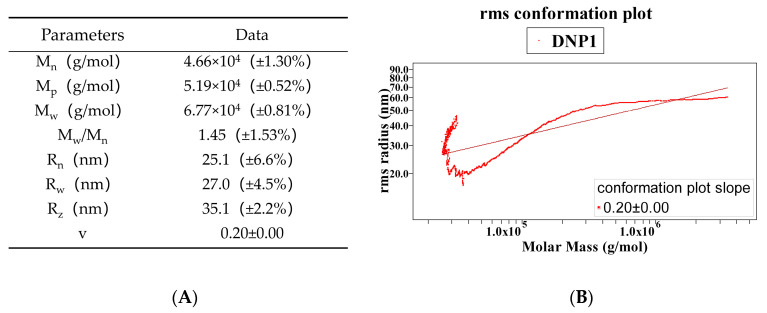
(**A**) Molecular weight and conformation parameters of DNP1, (**B**) conformation plot of DNP1 in 0.1 mol/L NaCl solution.

DNP1 was obtained after separation and purification. It was a glucomannan composed of Man (75.86 ± 0.05%) and Glc (24.14 ± 0.05%) [12]. The weight-averaged molecular weight (M_w_), number-averaged molecular weight (M_n_), root-mean-square radius (R_g_), and polydispersity (M_w_/M_n_) represent significant data for the characterization of polysaccharides. As shown in Figure 1A, the molecular weight of DNP1 was 6.77 × 10^4^ (±0.81%) Da, and the polydispersity coefficient (M_w_/M_n_) of DNP1 was 1.45 (±1.53%), indicating that DNP1 was a polysaccharide with wide distribution. It may be that the cavitation effect produced by ultrasound during the extraction process degraded DNP1 and produced a large number of small-molecule fragments, which caused a decrease in molecular weight and broadening of the distribution, leading to a larger polydispersity coefficient and a wider molecular weight distribution [28].

Due to composition, structure, chain length, and external force, polysaccharide molecules will curl to different degrees and show different chain morphology (such as rod chain, free spiral chain, or spherical chain), which is a unique property of macromolecular polysaccharides [29]. The radius of the root mean square is related to the mass distribution of polysaccharide molecules, which can measure the size of polysaccharide molecules. If the polysaccharide is a macromolecule with R_g,z_ greater than 10 nm, the relationship between R_g,z_ and M_w_ of the polysaccharide (R_g,z_ = KM_w_*^v^*) provides information about the conformation of the polysaccharide. When performing the bilogarithmic plot of log R_g,z_ vs. log M_w_, the slope (*v*) is 0.33, 0.5–0.6, and 1, the chain morphology is spherical, free spiral, and rigid rod, respectively. When the value of *v* is less than or equal to 0.33, the polymer molecules are compact and uniform spheres [30]. Figure 1B shows that the *v*-value of DNP1 is 0.20, indicating a spherical conformation.

### 3.2. Structural Analysis

In the DNP1 methylation results (Table 1), there were two main derivatives, that is, 1, 4, 5-tri-O-acetyl-2, 3, 6-tri-O-methyl mannitol and 1, 4, 5-tri-O-acetyl-2, 3, 6-tri-O-methyl glucitol, with a molar ratio of 3.17: 1. A small proportion of terminal groups were detected, which may be t-Man*p*. These results indicated that DNP1 is a glucomannan composed of →4)-β-Man*p*-(→1 and →4)-β-Glc*p-*(→1 without branching linkage. The methylation results were basically consistent with those of the monosaccharide composition.

**Figure 2:**
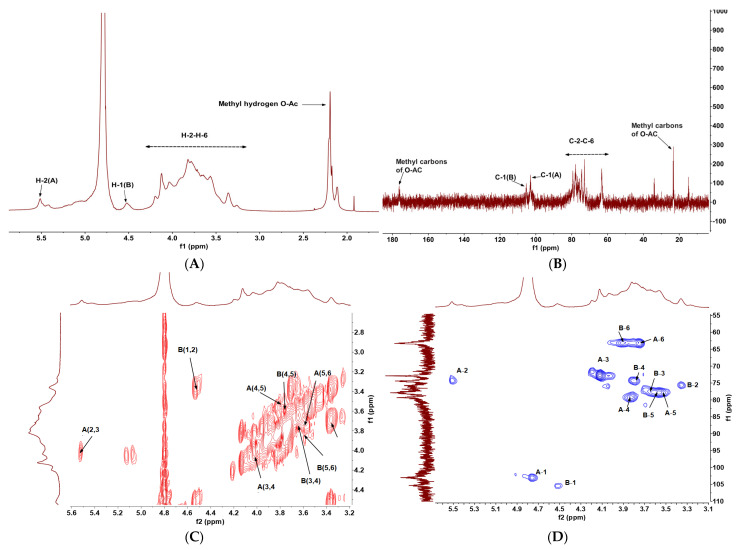

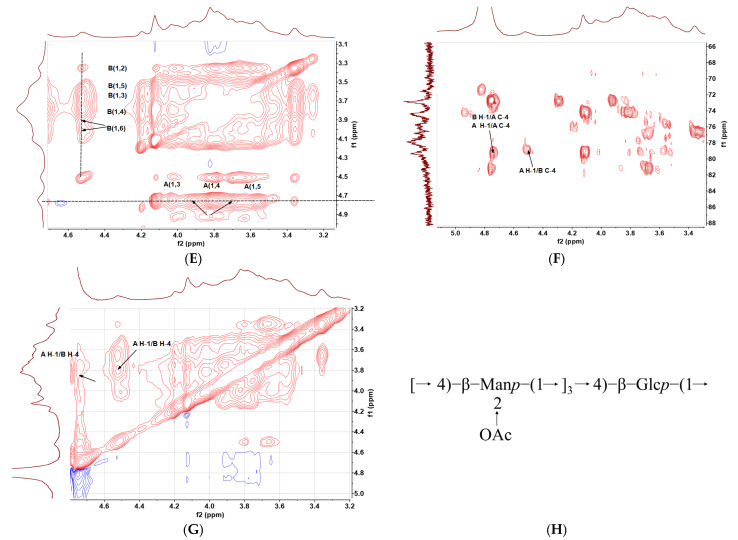
NMR spectra of DNP1: (**A**) ^1^H NMR, (**B**) ^13^C NMR, (**C**) COSY, (**D**) HSQC, (**E**) TOCSY, (**F**) HMBC, (**G**) NOESY, (**H**) the possible repeating structural units of DNP1.

The NMR spectra of DNP1, both one-dimensional (^1^H and ^13^C) and two-dimensional (including COSY, NOESY, HSQC, and HMBC), were studied and analyzed. Chemical shifts in β-type glycosides are known to be δ 4.4–5.0 ppm, and α-type glycosides are known to be δ 5.0–5.4 ppm [31]. The signal of the anomeric proton DNP1 was found to be around δ 5.0 ppm (Figure 2A), and there was a set of complex signals in the anomalous region (4.50–5.50 ppm), indicating that DNP1 was a heteropolysaccharide containing multiple sugar residues.

The signals of 1.91–2.21 ppm in the ^1^H spectrum (Figure 2A) and the signals of 23.11 ppm and 176.11 ppm in the ^13^C spectrum (Figure 2B) indicate the presence of O-acetyl in DNP1 [32]. According to the ^1^H spectrum, the ^13^C spectrum and the HSQC spectrum (Figure 2D) of DNP1, two anomeric proton signals were found at δ 4.75 ppm and δ 4.50 ppm, corresponding to 105.74 ppm and 102.93 ppm, labeled as residues A and B, respectively. ^13^C NMR has a wider range of chemical shifts than ^1^H NMR, which can not only determine the positions of various carbons but also distinguish the configuration and conformation of molecules [33].

We found that 4.75 ppm was the H-1 chemical shift of residue A and was observed in the HSQC spectrum (Figure 2D), which is a reasonable starting point for determining the H-2 to H-5 chemical shift. Some mannose residues have an acetyl group at the O-2 position [34]. The presence of O-acetyl-containing C in both mannose and glucose can substantially enhance the chemical shift in the proton that is directly attached to C, as well as the chemical shift in the proton. Therefore, the chemical shift at 5.51 ppm could be attributed to the H_2_ of OAc-β-Man*p*. On this basis, H-2/H-3 (5.51/4.03) and H-3/H-4 (4.03/3.83) were successfully inferred from the COSY spectrum (Figure 2C). Cross peaks at δ 4.75/3.55, 4.75/3.72, and 4.75/3.92 ppm in the TOCSY spectrum (Figure 2E) suggested H-5-H-6 of residue A. Therefore, cross peaks of 4.75/102.93, 5.51/74.24, 4.03/72.78, 3.83/79.40, 3.55/77.87, and 3.77, 3.92/63.00 ppm in the HSQC spectrum (Figure 2D) indicated the H-1/C-1-H-6/C-6 signals of residue A. Combined with monosaccharide composition, methylation results, and the previous literature, residue A was assigned to →4)-2-OAc-β-Man*p*-(→1 [34].

Indeed, 4.50 ppm was the H-1 chemical shift in residue B, and H-2 to H-6 were inferred from the cross peaks of H-1/H-2 (4.50/3.35), H-2/H-3 (3.35/3.65), H-3/H-4 (3.65/3.77), H-4/H-5 (3.77/3.57), and H-5/H-6 (3.57/3.92, 4.00) in the COSY (Figure 2C) and TOCSY (Figure 2E) spectra. According to the correlation peaks of chemical shifts in the HSQC spectrum, 105.74, 75.58, 77.07, 78.87, 77.99, and 63.00 ppm were assigned to C-1, C-2, C-3, C-4, C-5, and C-6, respectively [34]. The chemical shifts in residue B were supported by previously reported data, and residue B was attributed to →4)-β-Glc*p*-(1→ [34].

After assigning the ^1^H and ^13^C chemical shifts in all sugar residues (Table 2), HMBC and NOESY spectra were used to reveal the inter-molecular and intra-molecular linkage order of sugar residues (Figure 2F,G). In the HMBC spectrum (Figure 2F), intra-molecular coupling between H-1 of residue A and C-4 of residue A was observed at 4.75/79.40 ppm. Additionally, a cross peak occurred between H-1 and H-4 of residue A (A H-1/A H-4) in the NOESY spectrum. It was indicated that a repeat segment of →4)-2-OAc-β-Man*p*-(1 → 4)-2-OAc-β-Man*p*-(1→ was present.

There were two inter-molecular correlation peaks, H-1 of residue A and C-4 of residue B at 4.75/78.87 ppm, H-1 of residue B and C-4 of residue A (B H-1/A C-4) at 4.50/79.40 ppm in the HMBC spectrum (Figure 2F), indicating a linkage of A → B and B → A. These results were also confirmed in the NOESY spectrum (Figure 2G), such as correlation peaks of A H-1/B H-4 and B H-1/A H-4 at 4.75/3.77 ppm, 4.50/3.83 ppm, respectively.

Summarily, according to the monosaccharide composition, methylation results, and NMR data, the possible repeating units of DNP1 are A→ A → A →B (Figure 2H), which means [→4)-2-OAc-β-Man*p*-(1→]_3_→4)-β-Glc*p*-(1→.

### 3.3. SPR Results Analysis

**Figure 3:**
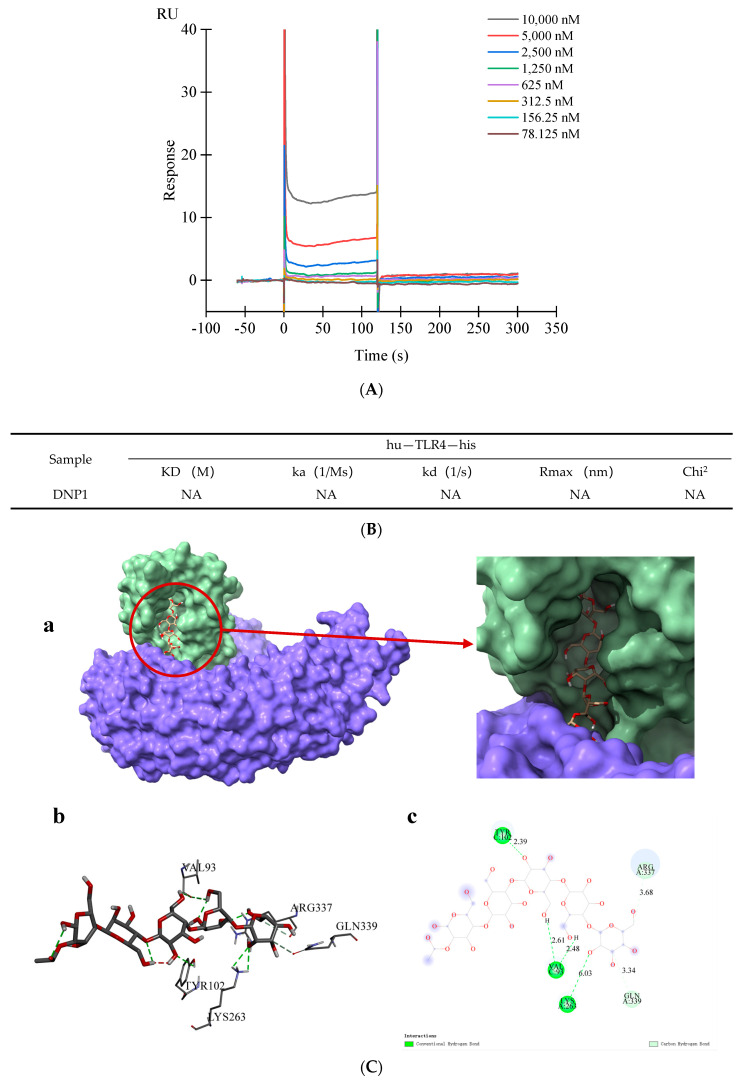
(**A**) SPR sensor diagram of DNP1 combined with TLR4, (**B**) binding kinetic parameters of DNP1 and TLR4 binding affinity by SPR analysis, (**C**) Molecular docking results between DNP1 and TLR4—MD2: (a) Molecular docking 3D model diagram, (b,c) DNP1 connects to VAL93, TYR102, LYS263, ARG337 and GLN339.

Molecular interaction technology has become an indispensable tool in the field of life sciences and has also made important contributions to the research on SARS-CoV-2 [35]. In recent years, SPR (surface plasmon resonance) technology has made progress in the screening and evaluation of the activity of polysaccharides, such as the use of SPR technology, to determine the interaction between sulfated polysaccharides and poly-l-lysine and evaluate their anti-HIV activity [36]. It is reported that TLR4 is the target protein of the immune activity of Dendrobium polysaccharide [37]. As shown in the binding kinetic parameters of SPR (Figure 3A) and sensor diagram (Figure 3B), the dissociation constant value of DNP1 was 0, which revealed that DNP1 has no binding force with TLR4, which indicated that DNP1 did not directly bind with TLR4 [35].

### 3.4. Molecular Docking

It has been reported that many natural compounds bind to TLR4-MD2 to exert pro-inflammatory or anti-inflammatory activities [24]. Therefore, to clarify the interaction between DNP1 and TLR4-MD2, a molecular docking assay was conducted. The active site of TLR4-MD2 is a pocket mainly composed of TLR4 domain residues and MD2 domain residues. Figure 3C showed that DNP1 was embedded in the active pocket formed by TLR4 and MD2 and inter-molecular interaction occurred. Figure 3C(a–c) show that DNP1 formed two hydrogen bonds with the VAL93 residue of TLR4 at a distance of 2.61 and 2.48, respectively, and formed a hydrogen bond with the TYR102 residue of TLR4 at a distance of 2.39. It formed a hydrogen bond with the LYS263 residue of MD2 at a distance of 6.03 and a carbon–hydrogen bond with the ARG337 residue of MD2 at a distance of 3.68. The binding energy is usually used to indicate the degree to which the ligand (DNP1) is bound to the target protein. A binding energy value less than 0 means that the ligand (DNP1) is free to bind to the target protein, and the smaller the binding energy value, the greater the likelihood of binding. The binding energy value of DNP1 to TLR4-MD2 was -7.9 kcal/mol, indicating that DNP1 could stably bind to TLR4-MD2. In terms of the binding energy and hydrogen bond, DNP1 can effectively and stably bind to TLR4-MD2.

### 3.5. Analysis of the Interaction Mode between DNP1 and TLR4

In our previous study, DNP1 was shown to have significant cell proliferation capacity in RAW264.7 and no cytotoxicity. DNP1 was able to significantly reduce NO and the inflammatory factors TNF-α, IL-1β, and IL-6 produced by LPS-stimulated RAW 264.7 [12]. Based on the above findings, in the present study, RAW 264.7 cells treated with the receptor blockers MD2-IN-1 or TLR4-IN-C13 were stimulated by LPS. The experiments were used to analyze the mode of action of DNP1 on TLR4 by measuring NO content and inflammatory cytokine levels.

#### 3.5.1. Cell Proliferation and Toxicity Analysis

Before investigating the in vitro anti-inflammatory effects of DNP1, it was crucial to evaluate the impact of DNP1 on cellular proliferation and cytotoxicity of RAW 264.7 cells after the administration of MD2-IN-1 and TLR4-IN-C13. The results showed that MD2-IN-1, TLR4-IN-C13, and DNP1 did not have significant effects on the proliferation and toxicity of RAW264.7 cells (Figure S1). The cell viability of the MD2-IN-1 and TLR4-IN-C13 treatments was 100.95 ± 3.94% and 100.62 ± 1.42%, respectively, when DNP1 was added at a concentration of 200 μg/mL. No significant differences in cell viability of RAW 264.7 cells were observed compared to the control group (*p* > 0.05). The findings indicated that DNP1 did not have a significant cell proliferative effect and cytotoxicity on RAW 264.7 within the set DNP1 concentration, regardless of the addition of MD2-IN-1 or TLR4-IN-C13.

#### 3.5.2. Effect of Receptor Blocking on NO

NO is an important inflammatory substance produced by macrophages to regulate the immune response. Macrophage RAW264.7 releases a large amount of NO after being activated by LPS. The previous anti-inflammatory activity showed that DNP1 could significantly reduce the large amount of NO released by RAW264.7 cells stimulated by LPS [12].

**Figure 4:**
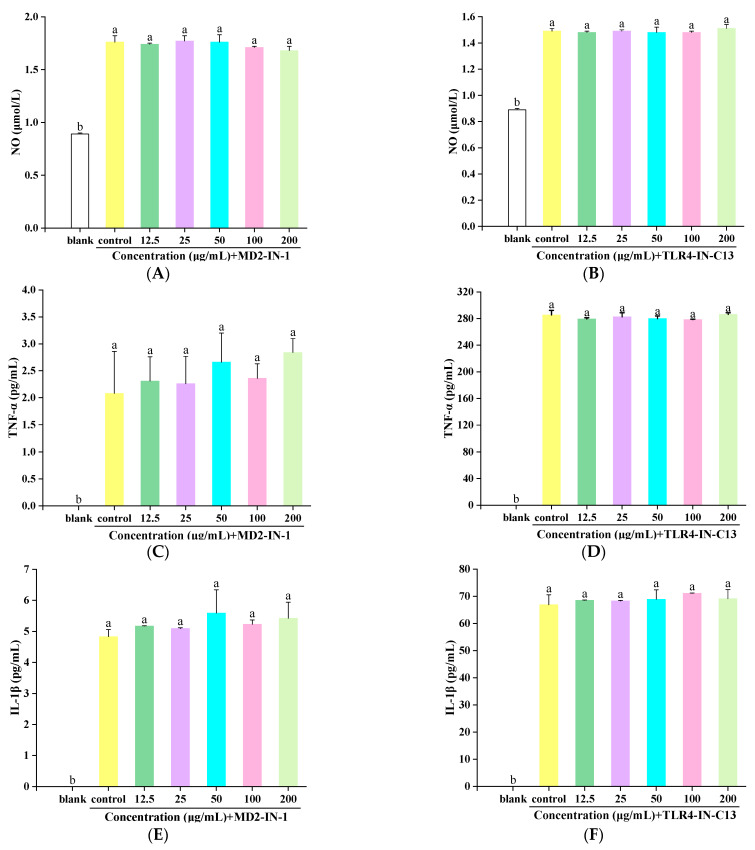

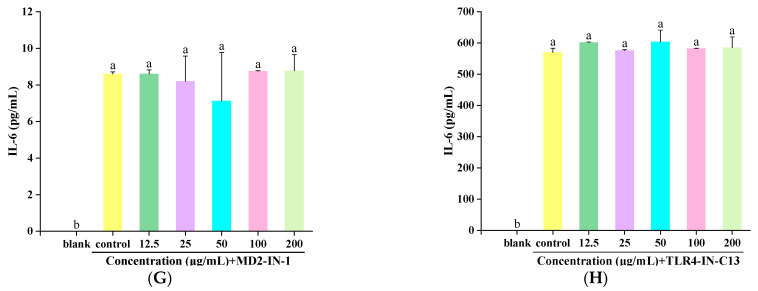
Effects of DNP1 on NO content in RAW 264.7 macrophages after adding MD2-IN-1 (**A**) and TLR4-IN-C13 (**B**), TNF-α content in RAW 264.7 macrophages after adding MD2-IN-1 (**C**) and TLR4-IN-C13 (**D**), IL-1β content in RAW 264.7 macrophages after addition of MD2-IN-1 (**E**) and TLR4-IN-C13 (**F**); IL-6 content in RAW 264.7 macrophages after adding MD2-IN-1 (**G**) and TLR4-IN-C13 (**H**). Significant differencaies are expressed by different letters (*p* < 0.05).

We added MD2-IN-1, and the effects of different concentrations of DNP1 on NO release from RAW 264.7 macrophages are shown in Figure 4A. After culturing with LPS (1 μg/mL), the NO content of RAW 264.7 macrophages in the TLR4-IN-C13 group increased significantly to 1.49 ± 0.02 μM. After the addition of DNP1 with the setting concentration, NO concentrations were calculated as 1.48 ± 0.01 μM, 1.49 ± 0.01 μM, 1.47 ± 0.04 μM, 1.48 ± 0.01 μM, and 1.51 ± 0.03 μM. The concentration of NO released by RAW 264.7 had no significant change (*p* > 0.05) after treatment with different concentrations of DNP1. The blocking results for the MD2-IN-1 group are shown in Figure 4B. After culture of RAW 264.7 with LPS (1 μg/mL), the NO content increased significantly, reaching 1.76 ± 0.06 μM. Then, for the addition of DNP1 with the setting concentrations, the NO concentrations were 1.74 ± 0.01 μM, 1.77 ± 0.05 μM, 1.75 ± 0.06 μM, 1.71 ± 0.01 μM, and 1.68 ± 0.04 μM, respectively. The concentration of NO released by RAW 264.7 had no significant change (*p* > 0.05) after treatment with different concentrations of DNP1. This indicated that DNP1 could not regulate NO release after inhibiting TLR4 or MD2.

#### 3.5.3. Effect of Receptor Blockage on Cytokines

The cellular cytokine levels are shown in Figure 4, where the blank group indicates the production of cytokines by RAW264.7 without the presence of LPS, and the control group indicates its production with LPS. Figure 4C shows that RAW 264.7 was cultured in the MD2-IN-1 group with LPS (1 μg/mL), and the TNF-α content increased to 2.08 ± 0.78 pg/mL. After the addition of DNP1 with setting concentrations, TNF-α contents were 2.31 ± 0.45 pg/mL, 2.26 ± 0.51 pg/mL, 2.66 ± 0.54 pg/mL, 2.36 ± 0.27 pg/mL, and 2.84 ± 0.26 pg/mL, respectively. The concentration of TNF-α released by RAW 264.7 had no significant change (*p* > 0.05) after treatment with different concentrations of DNP1. The results of the TLR4-IN-C13 group (Figure 4D) were similar to those of the MD2-IN-1 group. The results showed that DNP1 could not play a role in a reduction in the content of TNF-α after adding MD2-IN-1 or TLR4-IN-C13. Additionally, similar results of IL-1β and IL-6 contents were also found in the MD2-IN-1 or TLR4-IN-C13 treatment groups (Figure 4E–H). However, the addition of DNP1 had no significant effect on cytokines in the MD2-IN-1 group and the TLR4-IN-C13 group.

The secretion of cytokines in the MD2-IN-1 group and the TLR4-IN-C13 group was significantly different. Obviously, the secretion of cytokines in the MD2-IN-1 group was lower than that in the TLR4-IN-C13 group (Figure 4). After blocking TLR4 target protein, RAW 264.7 cells still secreted a large amount of cytokines; however, after blocking the MD2 target protein, cells barely secreted IL-1β and IL-6, and they only secreted a small amount of TNF-α. The reason for this difference may be that the structure of the selected TLR4 inhibitor is highly similar to LPS, and TLR4-IN-C13 can activate inflammatory pathways, so RAW 264.7 cells can still secrete a large number of cytokines after blocking the TLR4 protein alone [38].

#### 3.5.4. Mode of Interaction between DNP1 and TLR4

**Figure 5:**
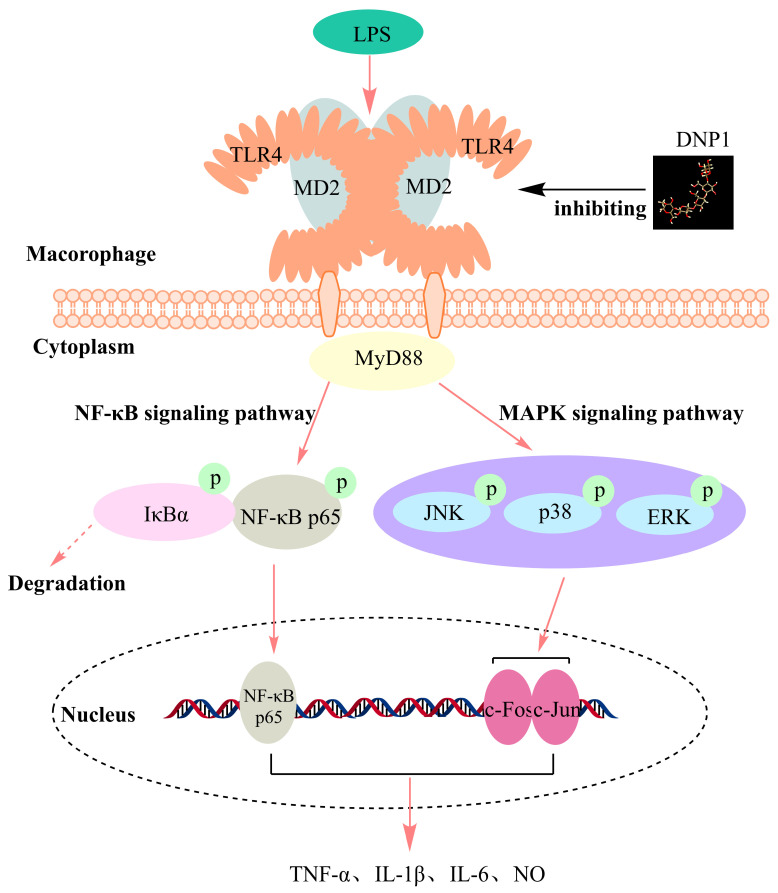
The mode of action of DNP1 on TLR4 and diagram of its anti-inflammatory pathway.

MD2 is a co-receptor for TLR4, and TLR4 must bind to MD2 before it can recognize LPS [39]. Upon binding to LPS, TLR4-MD2 forms a receptor multimer consisting of two TLR4/MD2/LPS complexes, which triggers downstream signaling to upregulate the expression of a range of inflammatory factors [40,41]. All experiments fully showed that DNP1 did not bind directly to TLR4. Instead, they required the mutual assistance of TLR4 and MD2 to exert their immune regulation activity. Inhibition of the MD2 or TLR4 target protein alone will make the anti-inflammatory activity of DNP1 unable to exert. Therefore, we speculate that DNP1 binds to TLR4 in a manner that may be consistent with the binding of LPS to TLR4. As shown in Figure 5, DNP1 regulates cellular immunity by binding to the TLR4-MD2 complex and causing structural changes in the TLR4-MD2 complex. This action inhibits the binding of LPS to the TLR4-MD2 complex and blocks the MAPK and NF-κB signaling pathways.

## 4. Conclusions

The molecular weight of DNP 1 was 6.77 kDa (±0.81%), with a spherical conformation in solution. The methylation results showed that DNP1 was composed of →4)-β-Man*p*-(→ 1 and →4)-β-Glc*p*-(→1 sugar residues. NMR spectra indicated that the possible repeating units of DNP1 were [→4)-2-OAc-β-Man*p*-(1→]_3_→4)-β-Glc*p*-(1→. The SPR results showed that DNP1 could not bind directly to the TLR4 target protein. Molecular docking simulated that DNP1 could bind to TLR4-MD2 stably. The macrophage MD2 and TLR4 receptor blocking assay verified that DNP1 needs MD2 and TLR4 to play a joint role in immune regulation. Therefore, the mode of binding of DNP1 to TLR4-MD2 may be consistent with that of LPS to TLR4-MD2; that is, DNP1 inhibits the binding of LPS to the TLR4-MD2 receptor by binding to the TLR4-MD2 complex and blocks the MAPK/NF-κB signaling pathway, thus regulating cell immune function.

## Figures and Tables

**Table 1:** Methylation analysis of DNP1.

Sample	Linkage Pattern	PMAA	Rt (min)	Ion Fragmentation (*m*/*z*)	Molar Ratio (%)
DNP1	t-Man*p*	1,5-di-O-acetyl-2,3,4,6-tetra-O-methyl mannitol	8.4	71, 87, 102, 129, 137, 145, 162, 191, 207, 218, 239, 260, 281, 301	4.711
	→4)-β-Man*p*-(1→	1,4,5-tri-O-acetyl-2,3,6-tri-O-methyl mannitol	12.5	71, 87, 102, 118, 129, 142, 162, 173, 233	72.460
	→4)-β-Glc*p*-(1→	1,4,5-tri-O-acetyl-2,3,6-tri-O-methyl glucitol	13.6	71, 87, 102, 118, 129, 142, 162, 207, 233, 260, 299	22.829

**Table 2:** ^1^H NMR and ^13^C NMR chemical shifts for DNP1 in D_2_O.

Residues	Chemical Shifts (ppm)						-OAc
	H-1/C-1	H-2/C-2	H-3/C-3	H-4/C-4	H-5/C-5	H-6/C-6	
→4)-2-OAc-β-Man*p*-(1→(A)	4.75 102.93	5.51 74.24	4.03 72.78	3.83 79.40	3.55 77.87	3.72, 3.92 63.00	1.91–2.21 23.11\176.11
→4)-β-Glc*p*-(1→(B)	4.50 105.74	3.35 75.58	3.65 77.07	3.77 78.87	3.57 77.99	3.92, 4.00 63.00	

## Data Availability

The original contributions presented in this study are included in the article/Supplementary Material; further inquiries can be directed to the corresponding author.

## Supplementary Materials

The following supporting information can be downloaded at: https://www.mdpi.com/article/10.3390/foods13091356/s1, Figure S1: Effects of different concentrations of DNP1 on proliferation of RAW 264.7 macrophages after adding MD2-IN-1 (A) and TLR4-IN-C13 (B); Figure S2: Total ion chromatogram of the partially methylated alditol acetates generated from DNP1, the mass spectra of specific partially methylated alditol acetates are shown below. In each mass spectrum the X axis denotes the m/z values whereas Y axis stands for relative abundance of the fragmented masses; Figure S3: The figure of homogeneity of DNP1.
