# Supplementary material for: Structure of Polysaccharide from Dendrobium nobile Lindl. and Its Mode of Action on TLR4 to Exert Immunomodulatory Effects
Source: Foods. 2024 Apr 28;13(9):1356. doi: 10.3390/foods13091356 (PMC11083282; doi:10.3390/foods13091356)

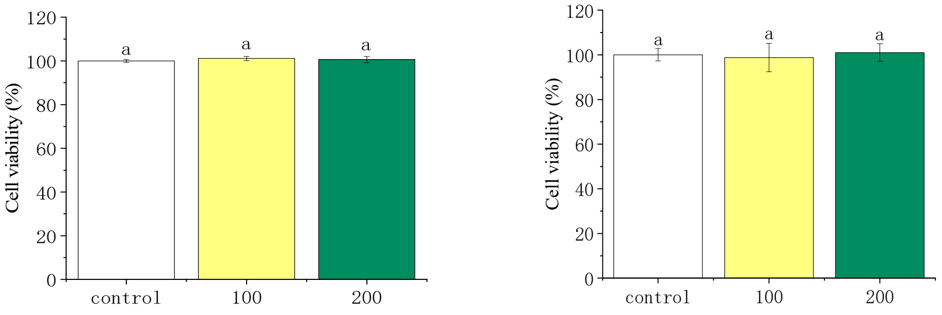

（A）　　　　　　　　　　（B）

Figure S1. Effects of different concentrations of DNP1 on proliferation of RAW 264.7 macrophages after adding MD2-IN-1 (A) and TLR4-IN-C13 (B)

Figure S2. Total ion chromatogram of the partially methylated alditol acetates generated from DNP1, the mass spectra of specific partially methylated alditol acetates are shown below. In each mass spectrum the X axis denotes the m/z values whereas Y axis stands for relative abundance of the fragmented masses.

Abundance

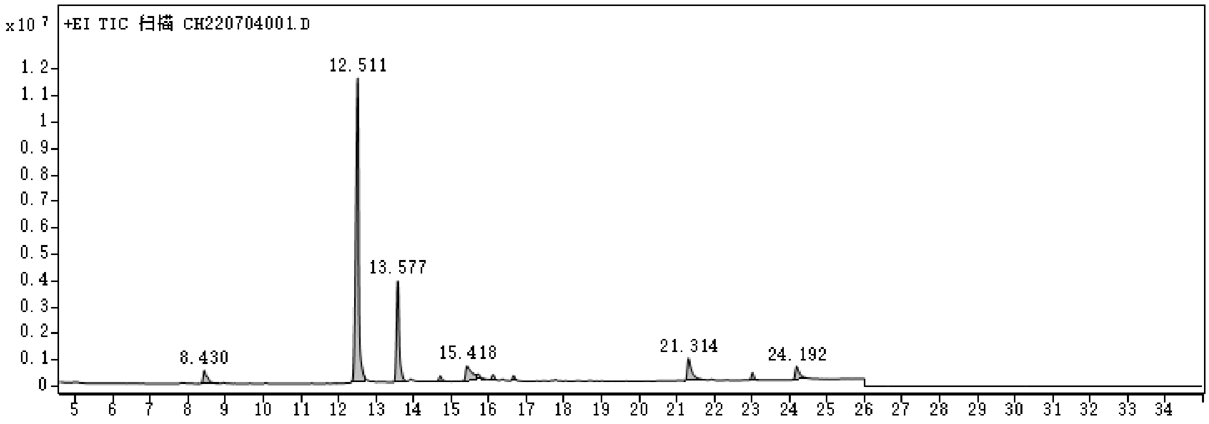

Time (min)

1. Mass spectrum of T-Man*p* (1,5-di-O-acetyl-2,3,4,6-tetra-O-methyl mannitol)

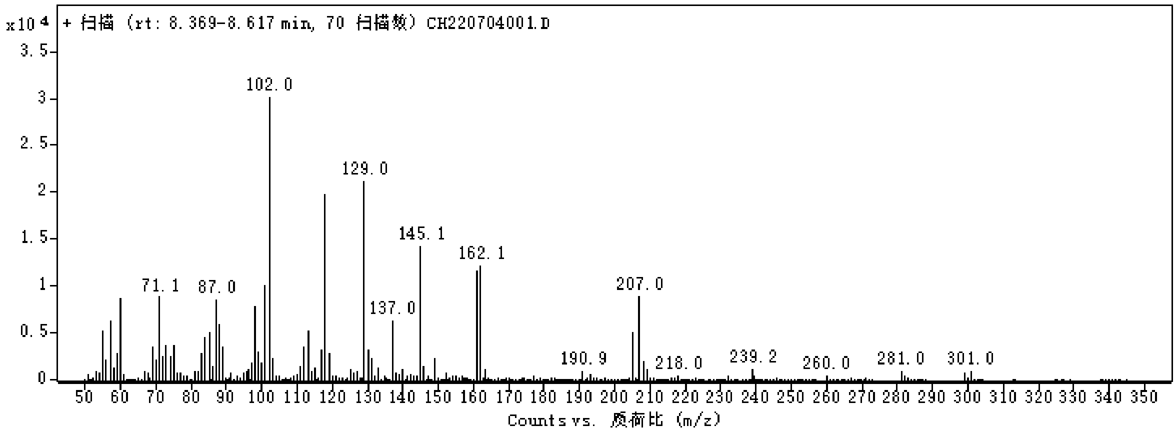

2. Mass spectrum of 1,4-Man*p* (1,4,5-tri-O-acetyl-2,3,6-tri-O-methyl mannitol)

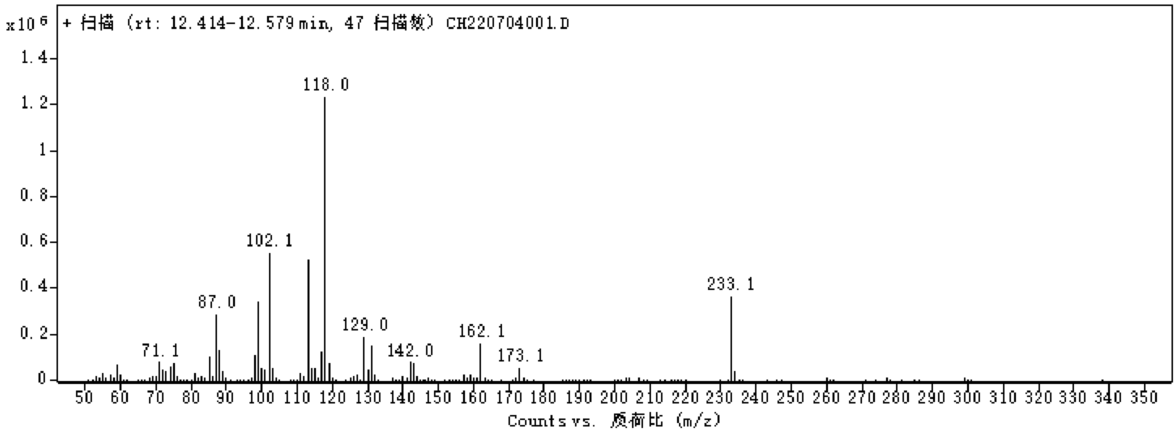

3. Mass spectrum of 1,4-Glc*p* (1,4,5-tri-O-acetyl-2,3,6-tri-O-methyl glucitol)

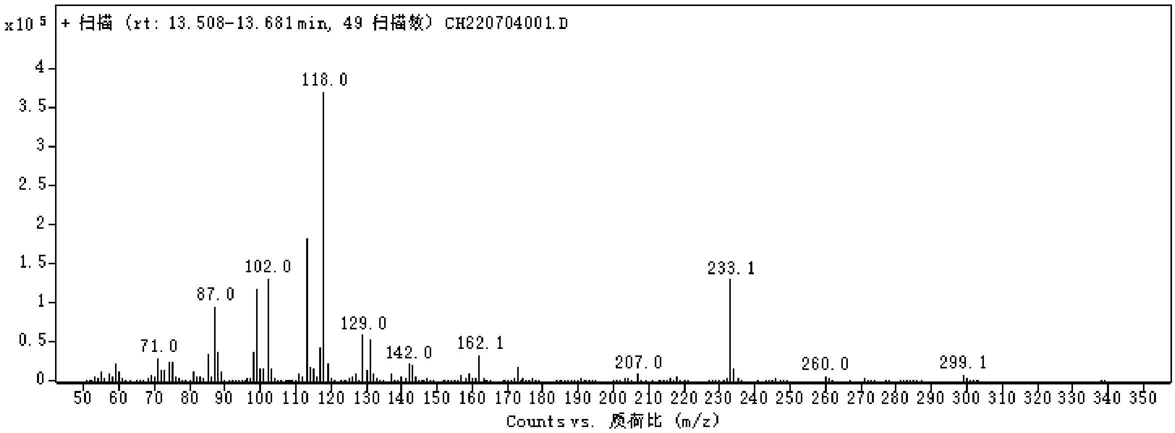

Figure S3. The figure of homogeneity of DNP1.

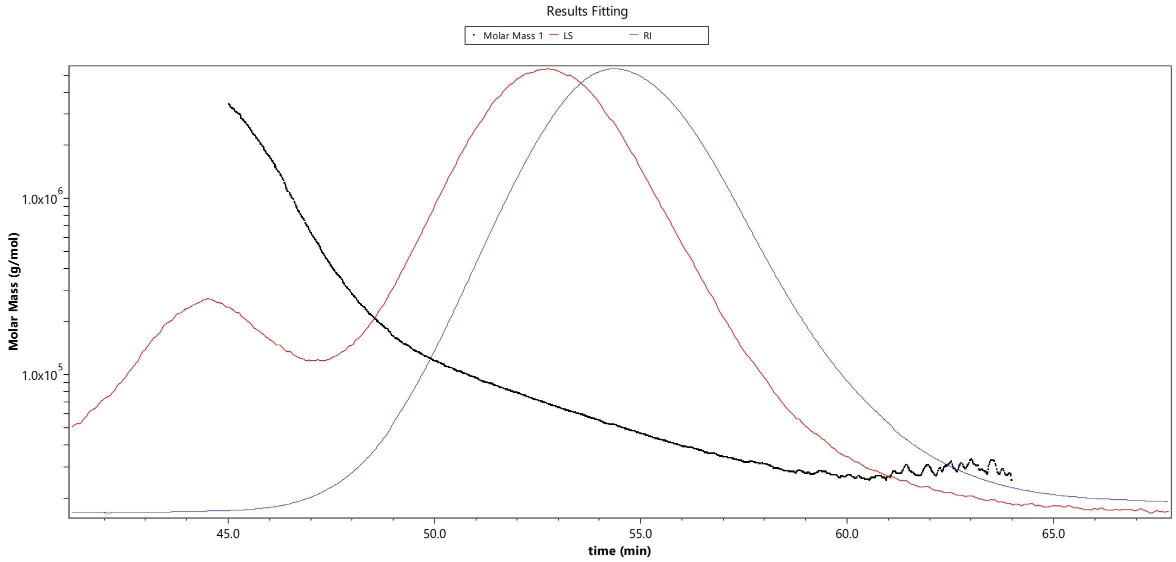

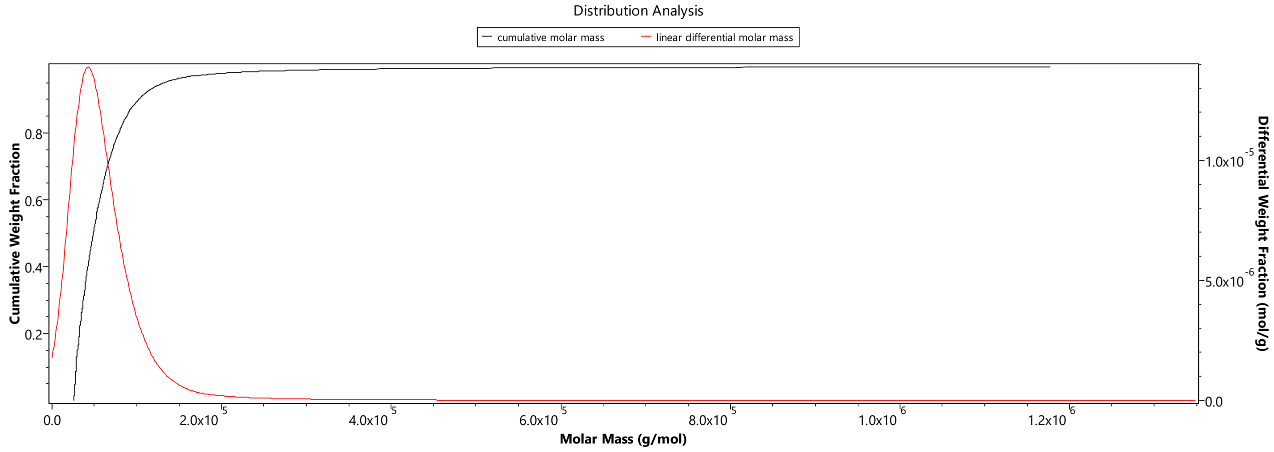

Supplement: Supplementary file 1 [file foods-13-01356-s001.zip › foods-2963203-supplementary.pdf]
